# Investigations on the potential of optical coherence tomography as an imaging tool for eustachian tube

**DOI:** 10.1038/s41598-021-87637-6

**Published:** 2021-04-13

**Authors:** Xiao-Mei Sun, Jia-Qi Luo, Zhi-Wen Xiao, Qing-Yu Gu, Lin-Chan Lan, Hui-Qing Zhang, Guan-Ping Zhang

**Affiliations:** Department of Otolaryngology Head and Neck Surgery, The Sixth Affiliated Hospital of Sun Yat-Sen University, Guangzhou, 510655 China

**Keywords:** Outcomes research, Medical research, Optics and photonics, Imaging, Optical spectroscopy, Structure determination

## Abstract

The purpose of this study was to explore the feasibility of eustachian tube optical coherence tomography (ET-OCT) for imaging the pharyngeal region of the eustachian tube (ET). Ten subjects with ear complaints underwent ET-OCT guided by nasal endoscopy, and ET-OCT examination was performed on both sides of each subject's ETs. The process and resulting images were analysed. Ten subjects ranging from 21 to 73 years old (45 ± 14.77) were enrolled in this study. Eighteen ET-OCT imaging examinations were completed. The mean duration of each examination was 2.80 ± 1.62 min (ranging from 2 to 7 min). There were no adverse events or complications. In some subjects, the ET-OCT images clearly presented the microstructures of the ET wall, including the lumen, mucosa, submucosa, cartilage and plica. However, in some subjects, it showed different characteristics, such as an unclear hierarchy and secretions in the lumen. ET-OCT may help to distinguish the structural composition of the ET and elucidate related pathophysiological mechanisms. It is a valuable imaging tool suited for the ET, with potential diagnostic value in determining the morphology of the lumen, intraluminal mucosa and submucosal tissue in the pharyngeal region of the ET.

## Introduction

Optical coherence tomography (OCT) is a non-invasive microscopic imaging technique that can provide cross-sectional images of tissue microstructures with high spatial resolution^1^. It can compensate for the shortcomings of microscopy and ultrasound in terms of transmittance and resolution, respectively. Due to its non-invasiveness, lack of radiation and high resolution, OCT is widely used in ophthalmology, pulmonology and gynaecology^2–4^. The endovascular diagnosis of coronary artery disease and tortuous cerebrovasculature of the brain by luminal OCT technology has also been well established^5–9^. However, there have been few reports on the clinical application of OCT in otology. Specialists have used OCT to evaluate the normal and pathological tympanic membranes of patients with secretory otitis media, showing that OCT can be used to obtain images of the tympanic membrane^10–13^. These studies represent the initial applications of OCT in the field of otology. Considering that OCT is a valuable imaging technology suitable for the observation of narrow lumens, we speculate that it can also be applied in the eustachian tube (ET).

The ET serves to equalize the air pressure of the middle ear with the atmospheric pressure^14,15^. In adults, the ET is approximately 31 ~ 39 mm in length. The lower 2/3 is the cartilage segment, and the upper 1/3 is the bony segment. The epithelial lining of the ET consists of pseudostratified ciliated columnar epithelium with goblet cells near the pharynx. Under the ET epithelial lining, the lateral region is filled with the ET glands, Ostmann’s fatty tissue (OF), connective tissue, cartilage, and the tensor veli palatini^16^.

Currently, evaluation of the ET is mainly focused on measuring the pressure of the ET and evaluating the patency of the ET, such as by tubomanometry (TMM), the Valsalva manoeuvre, impedance audiometry, the eustachian tube score (ETS), the nine-step test, sonotubometry and the eustachian tube dysfunction questionnaire-7 (ETDQ-7)^17,18^. Images obtained by imaging examinations, such as computed tomography (CT) and magnetic resonance imaging (MRI), cannot show the status of the mucous membranes^19–22^. Endoscopic evaluation of the ET based on edema of the ET torus, erythema of the ET torus, exudate at the ET orifice, and the presence of tubal tonsil is mainly focused on the score of the state of the ET orifice mucosa^23^. Biopsy is needed to obtain details regarding the tissue structure hierarchy, which may cause secondary ET dysfunction; thus, morphology research remains challenging. Understanding the luminal surface and the surrounding soft tissue of the ET will help in determining the aetiology of diseases associated with abnormal ET function. One study first used OCT to explore the application of OCT in examining the ET in sheep, and the results showed that the OCT images were highly correlated with the histological cross-sectional images, from the inner cavity to the ET^24^. Hence, as a non-invasive, real-time imaging method, OCT may be a promising option for ET inspection.

This study aimed to investigate the feasibility of ET-OCT via the eustachian orifices in humans and confirm that the images show the cross-sectional histological hierarchy of the ET wall, such as the mucosa, cartilage, and submucosa.

## Materials and methods

### Subjects

Patients with ear complaints who visited the Department of Otorhinolaryngology of the Sixth Affiliated Hospital of Sun Yat-Sen University between July and October 2020 were enrolled in this research before treatment. Clinical data acquisition and ET-OCT imaging were performed under a protocol approved by the Ethics Committee of the Sixth Affiliated Hospital of Sun Yat-Sen University (2020ZSLYEC-155) after informed consent was obtained from the patients.

### ET-OCT imaging

A commercial rigid nasal endoscope (TC200, Karl Storz, Germany) was used to collect high-resolution images of the nasal cavity and nasopharynx^23^. An ET insufflation catheter with an outer diameter of 4 mm and an inner diameter of 3 mm was used to guide the ET-OCT probe to the pharyngeal region of the ET correctly.

Scans were performed using an OCT imaging system (YSD-OCTIS-R-A1, Guangzhou Winstar Medical Technology Company Limited, Guangzhou, China)^25^. The system emitted a central wavelength of 1310 nm, with a measured lateral resolution of 25 μm and axial resolution of 15 μm. The probe (YSD-LC1715RA) was 1.7 mm in diameter and 1.5 m in length, and there was a 5-mm gap between the scanner and the tip.

### ET-OCT scanning protocol

**Figure 1:**
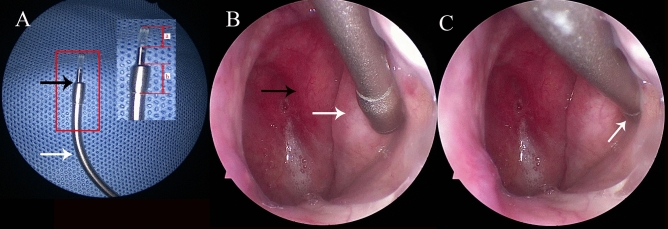
Insertion of the ET-OCT probe into the ET under the guidance of nasal endoscopy. (**A**) Insertion of the ET-OCT probe into the ET insufflation catheter. White arrow, ET insufflation catheter. Black arrow, ET-OCT probe; (a) gap between the light source and the tip (5 mm); (b) end of the ET insufflation catheter (5 mm). (**B**) Arrival of the ET-OCT probe at the pharyngeal orifice of the ET. White arrow, torus tubarius; black arrow, nasopharynx. (**C**) Insertion of the ET-OCT probe into the ET and initiation of image acquisition. White arrow, pharyngeal orifice.

Step 1: The subject's nasal cavity was preliminarily assessed under anterior nasal endoscopy for the presence of nasal septal deviation, nasal polyp hypertrophy and neoplasm, etc. Step 2: Furacillin ephedrine and tetracaine cotton tablets were used to contact the nasal mucosa and apply topical anaesthetic. Step 3: Routine examination of nasal and nasopharyngeal structures under rigid nasal endoscopy was performed. Step 4: The ET-OCT probe was placed into the ET insufflation catheter. Under the guidance of nasal endoscopy, the ET insufflation catheter with the ET-OCT probe was advanced to the pharyngeal opening of the ET and fixed. Then, the ET-OCT probe was gently and slowly inserted into the lumen of the ET until the operator felt resistance. ET-OCT scanning with uniform automatic retraction was begun, and continuous images were collected simultaneously until the probe was pulled back into the ET insufflation catheter. Step 5: The ET insufflation catheter, ET-OCT probe and rigid nasal endoscope were removed, completing the unilateral examination. Contralateral OCT scanning was performed in the same manner; examinations were first performed on the right and then on the left. During the test, if the subject felt pain or other discomfort or if the operator experienced difficulty placing the instrument, the ET-OCT examination on that side was terminated. The ET insufflation catheter and ET-OCT probe are shown in Fig. 1A. The positions of the ET insufflation catheter and ET-OCT probe when the ET-OCT probe arrived pharyngeal opening of the ET and was then inserted into the ET for OCT image acquisition are shown in Fig. 1B,C.

### OCT image processing and analysis

Images were generated at 10 frames per second, and every 30 frames was equivalent to 1 cm. The OCTIS software workstation version V1079 of the OCT device was used for image acquisition, processing and analysis. The imaging system can penetrate the ET mucosal surface to a depth of 2 mm, providing images with a resolution of 1024 × 1024 pixels. The SPSS version 20.0 software package (IBM Corporation, Armonk, NY, USA) was used for statistical analysis of the demographic characteristics and examination time. Data are expressed as the mean ± standard deviation. Categorical data are expressed as percentages (%).

## Results

### Patient characteristics

Ten subjects were enrolled in this study. The mean age was 45 ± 14.77 years (range 21–73 years). Bilateral ET-OCT imaging was performed in each subject. Eighteen ET-OCT imaging examinations were completed. The patient characteristics and reasons for examination failure are shown in Table 1. The mean duration of each OCT examination was 2.80 ± 1.62 min (range 2–7 min). There were no adverse events or complications, such as severe pain, bleeding, mucosal injury, or ear fullness. Both the tip and extending probe part of the ET insufflation catheter were included when calculating the depth of the probe into the ET. Our research showed that the average depth of the ET-OCT probe entering the ET lumen was 10.98 ± 0.48 mm (Table 2).

**Table 1:** Patient characteristics.

No	Sex	Age	Time (min)	Diagnosis	Characteristics		Reasons for NC
					R	L	
1	M	49	2	Left cholesteatoma	Normal	ET wall thickening	
2	M	26	2	Right cholesteatoma	ET wall thickening	Not completed	Septum deviated to left and the left nasal cavity is narrow
3	F	40	7	Bilateral chronic otitis media	Submucosal thickening	Blurred boundary	
4	F	44	2	Left chronic otitis media	Submucosal thickening	ET wall thickening and blurred boundary	
5	F	40	4	Left eustachian tube is dysfunctional	Normal	Mucosal thickening and layer unclear, secretion in lumen	
6	M	55	2	Bilateral chronic otitis media	Normal	Blurred mucosal layer	
7	M	50	2	Left chronic otitis media	Not completed	Normal	The septum deviated to right and the right nasal cavity is narrow
8	M	73	2	Right secretory otitis media	Submucosal layer thickening	Normal	
9	M	21	2	Chronic rhinitis, Epstein Barr virus positive	Normal	The mucous layer thinning	
10	F	52	3	Right chronic otitis media	Submucosal layer is unclear	Normal	

*NC* not completed.

**Table 2:** Summarized data of patient characteristics and ET-OCT scanning.

Characteristics	n
Age	45 ± 14.77 (21–73)
**Sex**	
Female	4 (40%)
Male	6 (60%)
Time of operation (min)	2.80 ± 1.62 (2–7)
Depth (mm)	10.98 ± 0.48 (10.20–11.77)
**Operation completion status of ET-OCT**	
No	2 (10%)
Yes	18 (90%)

### Characteristics of images obtained by ET-OCT for microstructural examination

**Figure 2:**
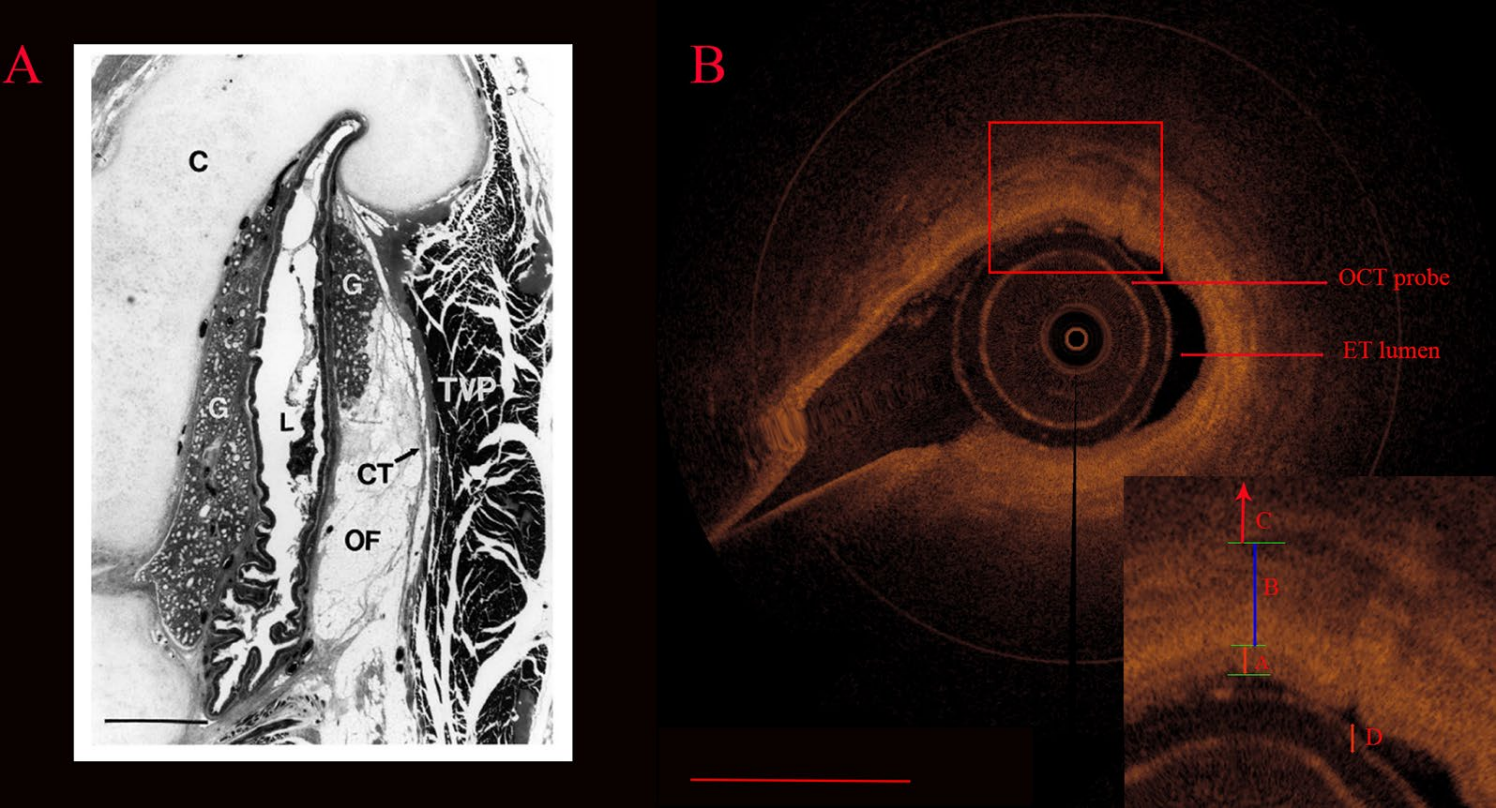
Image of the ET. (**A**) Photomicrograph of a vertically cut histologic section of the ET. *G* glands, *CT* connective tissue, *C* ET cartilage, *L* ET lumen, *TVP* tensor veli palatini muscle. Scale bar, 1 mm^16^. (**B**) OCT image of the ET. (**A**) mucosa; (**B**) submucosa; (**C**) cartilage, (**D**) plica. Scale bar, 1.7 mm.

ET-OCT images are illustrated in Fig. 2. The image quality was very good. According to the histological characteristics of ETs previously reported in the literature, we found that the OCT images clearly showed corresponding hierarchical characteristics. Clear hierarchical structures were observed, with a smooth ET wall and distinct lumen, mucosa, submucosa, cartilage and plica. The microstructures were clearly identified.

**Figure 3:**
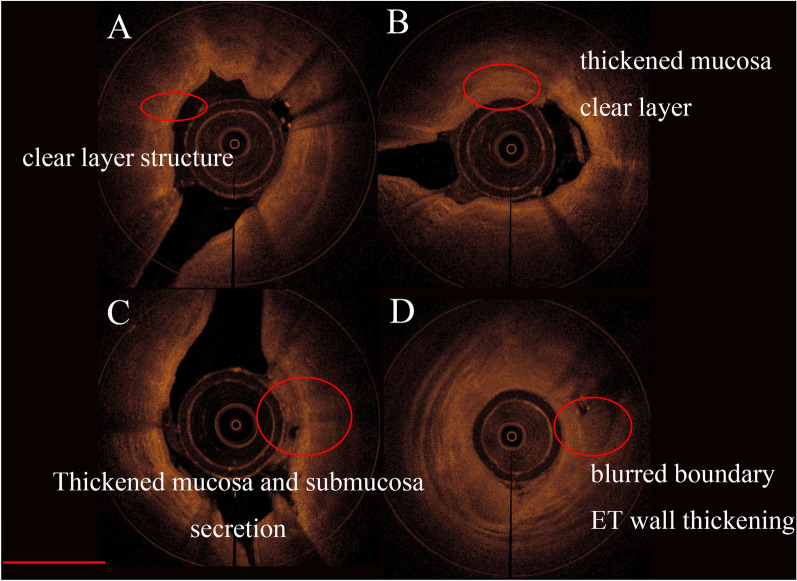
ET-OCT images with diverse characteristics. (**A**) Normal: mucosa, submucosa, and cartilage were well bedded. (**B**) Thickened mucosa and clear layer. (**C**) Thickened mucosa and submucosa with secretions in the ET lumen. (**D**) ET wall thickening and blurred boundary. Scale bar, 1.7 mm. SPSS version 20.0 https://www.ibm.com/support/pages/node/723799.

High-quality ET-OCT images showed clear hierarchical structures, with a smooth ET walls in normal subjects. However, some subjects showed different characteristics, such as an unclear hierarchy, secretions in the lumen, mucosal thickening, submucosal thickening, and ET wall thickening (Fig. 3).

## Discussion

In our study, ET-OCT was used to investigate the microstructures of the ET. The ET-OCT images clearly presented the hierarchical structures of the mucosa, submucosa, plica and cartilage, replicating the previously reported characteristics of histological sections. We applied nasal endoscopy to navigate the ET-OCT probe to the ET accurately in humans for the first time, with successful results. Furthermore, the probe could enter the ET to a maximum depth of 10.20 ~ 11.77 mm. According to the anatomical characteristics of the ET, the ET-OCT probe was mainly located in the cartilage segment and did not reach the isthmus of the ET. Therefore, the ET-OCT images presented the structures of the cartilage ET segment. Some patients complained of mild, tolerable swelling and pain without bleeding or other side effects. Furthermore, due to the short recording time, subjects were able to undergo the ET-OCT examination accompanied by nasal endoscopy under topical nasal anaesthesia. However, in patients with a deviated septum and a narrow nasal cavity on one side, the probe could not be placed in the ET because the inspection procedure could induce pain. Therefore, subjects with narrow nasal cavities are not suitable candidates for ET-OCT.

The ET-OCT images showed diversified characteristics in this study. Li et al. performed a point-by-point comparison of endobronchial optical coherence tomography (EB-OCT) images and pathological findings in an animal model of tracheal stenosis and showed that EB-OCT could distinctly display morphological abnormalities of the mucosal and submucosal layers and airway cartilage^25^. According to human small airway research, OCT can produce high-resolution images of the airway morphology, with good correlations with CT and histopathological examination results^3,14,26–28^. Based on the experience of identifying the characteristics of OCT images from these previous studies, we found that some ET-OCT images presented characteristics similar to those of airways, such as an unclear hierarchy, secretions in the lumen, mucosal thickening, and ET wall thickening. Images presenting an unclear hierarchy were similar to those of the mucous membrane fibrosis caused by chronic inflammation. ET wall thickening with a clear hierarchy may correspond to mucosal oedema with acute inflammatory changes. ET-OCT imaging can provide differentiated images reflecting the morphological features of the ET. This new approach may expand the application of OCT to include ET dysfunction and disease.

To date, there are no reliable methods for definitively evaluating the ET in clinical practice. TMM, the ETDQ-7, otoscopy, nasal endoscopy, temporal bone CT and acoustic impedance tests are generally required for comprehensive judgement^17,23^. These examinations mainly emphasize the functional opening movement and subjective feeling and do not involve information about the state of the mucosa or ET lumen wall. Our study shows that ET-OCT can provide high-resolution images of the ET wall and the clear structural hierarchy. Meanwhile, diverse image characteristics were also observed. These new findings may indicate the real state of the ET. Investigating ET wall morphological abnormalities can further our understanding of the mechanism of different types of ET dysfunction and disease. This approach could also help clinicians make proper decisions regarding therapy.

## Conclusion

ET-OCT may be helpful in distinguishing the morphological composition of the ET and in elucidating related pathophysiological mechanisms. It is a valuable imaging tool suited for the ET, with potential diagnostic value in determining the morphology of the lumen, mucosa and submucosa in the pharyngeal region of the ET.

Based on our research, ET-OCT is an imaging tool with the potential to facilitate the assessment of ET function. Although further clinical trials are needed, this groundwork shows that ET-OCT can complement or even potentially replace the current examination methods. Further study in a large sample with quantitative measurements is warranted. Further researches on comparing the images from the infected ET and healthy subjects and establishing the layer identification protocols in ET-OCT images remain further work. The automatic identification of tissue characteristics based on optical attenuation coefficients, machine learning algorithms, and deep learning techniques can improve the prospects of ET-OCT.
